# Return to On-Snow Performance in Ski Racing After Anterior Cruciate Ligament Reconstruction

**DOI:** 10.1177/03635465241307212

**Published:** 2025-01-20

**Authors:** Nathaniel Morris, Ricardo da Silva Torres, Mark Heard, Patricia Doyle Baker, Walter Herzog, Matthew J. Jordan

**Affiliations:** † Integrative Neuromuscular Sport Performance Laboratory, Faculty of Kinesiology, University of Calgary, Calgary, Alberta, Canada; ‡ Human Performance Laboratory, Faculty of Kinesiology, University of Calgary, Calgary, Alberta, Canada; § Artificial Intelligence Group, Wageningen University & Research, Wageningen, the Netherlands; ‖ Department of ICT and Natural Sciences, Norwegian University of Science and Technology, Ålesund, Norway; ¶ Banff Sport Medicine Clinic, Canmore, Alberta, Canada; # School of Medical and Health Sciences, Edith Cowan University, Joondalup, Western Australia, Australia; Investigation performed at University of Calgary, Calgary, Alberta, Canada

**Keywords:** alpine ski racing, knee injury, anterior cruciate ligament, return to performance

## Abstract

**Background:**

The individual variation in on-snow performance outcomes after anterior cruciate ligament (ACL) reconstruction (ACLR) in elite alpine ski racers has not been reported and may be influenced by specific injury characteristics.

**Purpose:**

To report the performance statistics of elite ski racers before and after ACLR and to identify surgical and athlete-specific factors that may be associated with performance recovery.

**Study Design:**

Descriptive epidemiological study.

**Methods:**

International Ski and Snowboard Federation (FIS) points, FIS ranking, average placing, and average percentage behind the winning time in each race were calculated for 30 national and provincial team ski racers at 1 year before and 3 years after ACLR. Injury characteristics were obtained from operative reports and clinical notes.

**Results:**

The mean age at the time of primary ACLR was 21.6 ± 3.5 years. Overall, 27 of 30 (90%) ski racers returned to the same preinjury competition level. Yet, only 16 of 30 (53%) improved in FIS points, and 13 of 30 (43%) improved in FIS ranking, in one of the speed or technical disciplines by 3 years after surgery. Of the skiers who improved in FIS points, 36% sustained multiligamentous injuries, 45% sustained meniscal tears, and 45% sustained chondral lesions. Of those who failed to improve in FIS points, 50% sustained multiligamentous injuries, 50% sustained meniscal tears, and 60% sustained chondral lesions. Meniscal tears and chondral lesions occurred mostly on the lateral side. The medial collateral ligament was involved in 8 of 9 multiligamentous injuries.

**Conclusion:**

These findings suggest significant individual variation in the recovery of on-snow performance of ski racers after ACLR, despite returning to the same competition circuit. The pattern of secondary injuries alongside primary ACL ruptures showed little association with improved performance.

An anterior cruciate ligament (ACL) rupture is the most common severe injury experienced by competitive alpine ski racers, with approximately 5.5 ACL injuries per 100 athletes per season at the World Cup level.^3^ An ACL injury frequently coincides with additional knee ligament injuries, meniscal tears, and chondral lesions,^17^ leading to significant time loss from racing.^7^ After an injury, ACL reconstruction (ACLR), followed by an extensive rehabilitation program, is often required for ski racers who intend to return to their preinjury level of function and competition.^15,18^

Previous reports have suggested that a high proportion of ski racers who sustain an ACL injury are able to return to their preinjury competition level (eg, World Cup, North American Cup) after surgical reconstruction^6,13^ compared with nonskiing athletes. However, a return to preinjury competition levels does not equate to a restoration of performance per se.^20^ For example, professional basketball players show significant decreases in performance statistics (eg, player efficiency rating, points per game, field goal percentage) in the first season after ACLR and tend to return to baseline within 2 to 3 years.^14,19,28^ Similarly, despite returning to competition around 7 months after ACLR, performance indicators in professional male soccer players can remain depressed until 2 to 3 years after surgery, suggesting that the return to performance process extends far beyond returning to sport.^2,21^ The current scientific literature suggests that returning to the same competition level, category, or circuit may not equate to restoration of the same preinjury performance level. This highlights the importance of quantifying return to performance statistics to better understand the effect of an ACL injury on sport performance and to better understand, on average, when or if alpine ski racers return to preinjury performance after an ACL injury. Within high-performance sport organizations, establishing expectations of performance outcomes after ACLR may influence decisions regarding surgery (eg, surgery timing, autograft choice), rehabilitation and return to sport, and funding support.^30^

Quantifying the effect of an ACL injury on alpine ski racing performance is challenging, given that ski racing is a time-based sport that occurs under constantly changing race conditions. The International Ski and Snowboard Federation (FIS) point system (FIS points) addresses this problem by standardizing race results across all competition circuits,^6,13^ but additional performance statistics may also help to further explain the effects of an ACL injury on ski performance. For example, preinjury and postinjury race data can be obtained from online databases, allowing season average performance statistics to be calculated for a given competition level. This can be used to quantify factors such as how far back a skier was from the winning time for races skied before and after the ACL injury at a given competition level (ie, average percentage behind the winning time) or his or her average placing before and after the ACL injury in a given season at a given competition level. These statistics may provide an additional complementary layer to the FIS point system that expands the current understanding of the effects of an ACL injury on alpine ski racing performance.^6,13^ Such analysis may also help provide important benchmark data for nonexperts in alpine ski racing to broadly understand how an ACL injury affects return to performance outcomes in competitive athletes, which is a crucial problem for competitive and elite athletes alike.^1,20^

A more expansive characterization of the preinjury performance level may also facilitate the use of targeted statistical approaches (eg, causal inference vs descriptive comparison) to investigate the effect of an ACL injury on performance.^26^ Such approaches depend entirely on the formulation of the research question.^26^ For example, “What is the effect of an ACL injury on the performance of a ski racer after they return to sport?” is a causal question that necessitates the use of causal inference methods. Here, an estimation of the effect of an ACL injury on skiing performance should consider the athlete’s previous performance trajectory (ie, how the skier was improving season over season before his or her injury) compared with the performance trajectory after the ACL injury using an estimation of what his or her progression would have been had he or she not been injured. Simply stated, did the skier end up achieving performance after the ACL injury that he or she would have achieved without an ACL injury? Propensity score matching is another statistical method that allows control participants with similar features to be identified and used as an appropriate benchmark to answer this “what if” question.^5,24^ Alternatively, descriptive comparisons can be used by formulating a question such as the following: “What is the likelihood that ski racers achieve the same skiing performance after an ACL injury compared with before an ACL injury?” Both questions are relevant for the return to performance evaluation of skiers after an ACL injury and may provide valuable information to sport organizations and practitioners working with skiers who sustain an ACL injury.

The scientific literature investigating on-snow performance measures in ski racers after ACLR is emerging and can assist in formulating better questions to support performance analysis in skiers with an ACL injury.^6,13^ Further, the association of prognostic factors such as age, injury severity, and competition level with the recovery of on-snow performance after ACLR is unclear. To address these gaps, we undertook a descriptive analysis of on-snow performance statistics in ski racers before ACLR and after ACLR to answer the following specific questions: (1) What is the individual variation in performance outcomes among competitive ski racers after ACLR, and do skiers after ACLR achieve a similar postinjury performance trajectory compared with control skiers identified with propensity score matching? (2) How does the use of different on-snow performance statistics compare as indicators of performance outcomes? (3) Are there surgical and athlete-specific factors that are associated with performance outcomes after ACLR?

## Methods

This study is a retrospective analysis of race results from subelite and elite alpine ski racers from 2005 to 2023 who had undergone ACLR. A performance database of national-level Canadian alpine skiers who underwent yearly testing at the same training center was accessed to generate a comprehensive list of athletes competing between 2005 and 2023. With the assistance of orthopaedic surgeons who had performed the ACLR procedures, medical team personnel, and sport administration personnel, this list was cross-referenced to identify skiers who sustained an ACL injury between 2005 and 2023 and subsequently underwent ACLR. Participants were contacted and provided written informed consent (University of Calgary Research Ethics Board [REB14-2270]), and operative reports were obtained from index ACLR. Male (n = 16) and female (n = 14) ski racers who were a minimum of 3 years after surgery were included. Athlete- and injury-specific data including age, sex, date of primary ACLR, limb that was injured, graft type, and date of revision or contralateral ACLR were obtained from clinical notes and operative reports. While rehabilitation was typically performed at a centralized training center, the postoperative care procedures and rehabilitation programs were unavailable for analysis.

On-snow performance data were extracted from the FIS database and included competition performance statistics before and after ACLR. Outcome measures included race date, location, discipline, competition category, finish time(s), race finish position, and FIS points (a global standardized scoring system used by FIS to measure race performance across performance levels and competition circuits). Disciplines were categorized as technical (giant slalom and slalom) or speed (downhill and super-G). Aligned with previous research,^13^ FIS points provided a standardized measure of alpine skiing performance, allowing global benchmarking of alpine ski racers in which lower FIS points indicate a higher ranking skier. The season-best (ie, lowest) FIS points of each skier for each discipline for each season before and after ACLR were calculated. FIS ranking for each skier in each discipline was also obtained for each season before and after ACLR. Finally, average placing (average race rank position) for each skier in each discipline in each race category was calculated for the season, along with the average percentage behind the winning time calculated from each race. Using the operative reports, any concurrent injuries present alongside primary ACL ruptures and graft type were also obtained and coded for analysis.

A control group without ACLR was formed from the cohort of ski racers by propensity score matching to the ACLR group based on sex, age at the time of injury, and FIS points at the time of injury. A 1:1 nearest neighbor matching method was used (‘MatchIt’ package in R). Low standardized mean differences of the covariates (absolute values <0.07) indicated balanced matches of ski racers with ACLR and control ski racers.

### Statistical Analysis

Athlete and injury characteristics are presented as the mean ± standard deviation or number (percentage). Performance statistics were calculated from the season before the ACL injury and at 3 years after surgery. Performance statistics of the control group were calculated at the age when matching occurred (referred to as the index age) and in the third year after the index age. A paired-samples *t* test was used to compare the mean values of performance statistics before and after ACLR. An independent *t* test was used to compare the mean age at the time of injury between male and female athletes. An independent *t* test was also used to compare the mean age at the time of injury between skiers who improved their performance after ACLR and skiers who did not. All analyses were conducted using RStudio (Version 4.2.3).

## Results

Participant characteristics are shown in Table 1. In the year before ACLR, 83% (25/30) of participants competed in the speed discipline, and 93% (28/30) competed in the technical discipline. The mean age at the time of primary ACLR was 21.6 ± 3.5 years. Overall, 27 of 30 skiers returned to the same level of competition (ie, World Cup, North American Cup, FIS), and 5 of 30 skiers progressed from the North American Cup circuit to the World Cup circuit after ACLR. In the technical discipline, ski racers completed 18.0 ± 11.6 races in the year before ACLR and 17.2 ± 8.7 races in the third year after ACLR. In the speed discipline, 8.6 ± 5.0 races were completed in the year before ACLR and 13.8 ± 7.7 races in the third year after ACLR.

**Table 1:** Participant Characteristics by Sex^*a*^

	Male (n = 16)	Female (n = 14)
Age at first ACLR, y	23.2 ± 3.0	20.5 ± 3.6
Highest competition category before ACLR		
World Cup	9 (56)	5 (36)
North American Cup	5 (31)	8 (57)
FIS	2 (13)	1 (7)
Graft type		
Hamstring tendon autograft	12 (75)	12 (86)
Bone–patellar tendon–bone autograft	00 (0)	00 (0)
Quadriceps tendon autograft	1 (6)	00 (0)
Allograft	3 (19)	2 (14)
Multiligamentous injury^*b*^		
Yes	5 (50)	4 (36)
No	5 (50)	7 (64)
Meniscal injury^*b*^		
Yes	7 (70)	8 (73)
No	3 (30)	3 (27)
Chondral injury^*b*^		
Yes	6 (60)	4 (36)
No	4 (40)	7 (64)
ACL reinjury (within 3 y after ACLR)	00 (0)	2 (14)
Contralateral injury (within 3 y after ACLR)	1 (6)	1 (7)

a Data are presented as mean ± SD or n (%). ACLR, anterior cruciate ligament reconstruction.

b Data are only available for 10 male and 11 female skiers.

**Figure 1.**
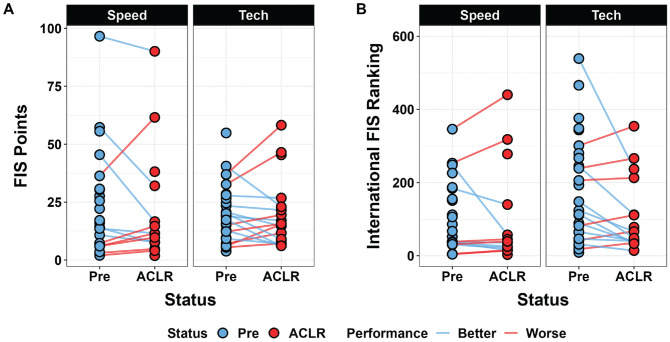
Pairwise comparisons of (A) International Ski and Snowboard Federation (FIS) points and (B) FIS ranking before and after anterior cruciate ligament reconstruction (ACLR) across disciplines. Lower FIS points indicate better performance. No data point for ACLR status indicates that the skier was not racing in the FIS circuit at this time point.

Additionally, by 3 years after ACLR, 3 of 30 participants had retired from racing. In the technical discipline, 39% (11/28) of ski racers had improved FIS points by 3 years after ACLR (Figure 1A). In the speed discipline, 32% (8/25) of ski racers had improved FIS points by 3 years after ACLR. Overall, 53% (16/30) of ski racers improved in FIS points in 1 of the 2 disciplines.

Consistent with FIS points, 39% (11/28) of ski racers achieved better FIS ranking in the technical discipline by 3 years after ACLR, but only 16% (4/25) of ski racers achieved higher FIS ranking in the speed discipline (Figure 1B). Overall, 43% (13/30) of ski racers had lower FIS ranking in 1 of the 2 disciplines. In ski racers competing at World Cups (n = 14), there were no differences before ACLR versus after ACLR in FIS points (7.0 ± 3.9 vs 5.6 ± 3.3, respectively; *P* = .25) or FIS ranking (27.4 ± 18.2 vs 21.8 ± 15.7, respectively; *P* = .32).

**Figure 2.**
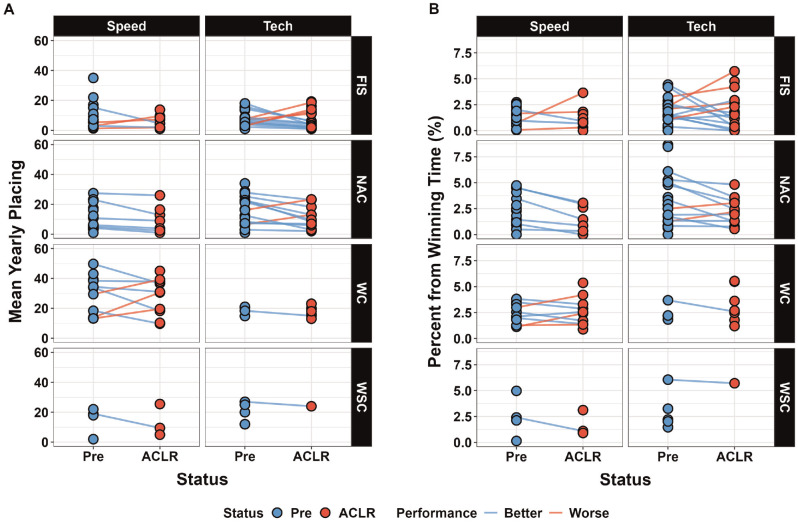
Pairwise comparisons of (A) average placing and (B) average percentage behind the winning time before and after anterior cruciate ligament reconstruction (ACLR) across disciplines and race categories. NAC, North American Cup; WC, World Cup; WSC, World Ski Championships.

Figure 2A shows the average placing for each participant across disciplines and race categories before and after ACLR. There were 12 skiers who competed in World Cup races in the speed discipline before ACLR, and 50% (6/12) improved their average placing at 3 years after ACLR (Table 2). In the technical discipline, 6 ski racers competed in World Cup races before ACLR, and only 33% (2/6) improved their average placing after ACLR.

**Table 2:** World Cup Skiers Who Improved Performance After ACLR^*a*^

Discipline	FIS Points	FIS Ranking	Average Placing	Average Percentage Behind Winning Time
Speed (n = 12)	6 (50)	3 (25)	6 (50)	5 (42)
Technical (n = 6)	2 (33)	2 (33)	2 (33)	2 (33)

a Data are presented as n (%). ACLR, anterior cruciate ligament reconstruction; FIS, International Ski and Snowboard Federation.

However, the other 4 skiers stopped competing at World Cup events altogether after ACLR. Figure 2B shows the average percentage behind the winning time for each skier across disciplines and race categories before and after ACLR. Of the skiers competing at the World Cup level in the speed discipline, 42% (5/12) decreased their average percentage behind the winning time. In the technical discipline, 33% (2/6) of the ski racers competing at World Cup before ACLR decreased their average percentage behind the winning time, with 3 of 6 in this group retiring within 3 years.

**Figure 3.**
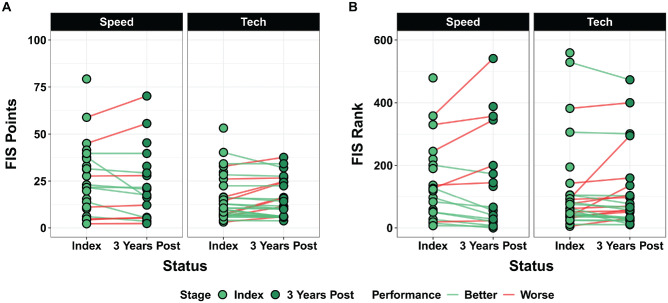
Pairwise comparisons of (A) International Ski and Snowboard Federation (FIS) points and (B) FIS ranking for the control group identified through propensity score matching at the index age and at 3 years after the index age.

In the control group, 60% (18/30) of the ski racers achieved lower FIS points, and 63% (19/30) of the ski racers had lower FIS ranking in 1 of the 2 disciplines in the third year after the index age (Figure 3).

Details of concurrent abnormalities, including multiligamentous injuries, chondral lesions, and meniscal tears, were available for 21 of the 30 skiers (Table 3). In all, 9 of the 21 (43%) skiers sustained multiligamentous injuries, 10 of 21 (48%) skiers sustained chondral lesions, and 15 of 21 (71%) skiers sustained meniscal tears. Of the 21 skiers, 11 (52%) achieved lower FIS points by 3 years after ACLR. Of the 9 ski racers with multiligamentous injuries, 4 (44%) achieved lower FIS points by 3 years after ACLR. Additionally, 4 of the 30 skiers experienced a second ACL rupture within 3 years of ACLR, with 2 of them sustaining an injury to the contralateral knee. All 4 skiers who experienced a second ACL rupture had received a hamstring tendon autograft.

**Table 3:** Surgical Characteristics by Improvement in FIS Points by 3 Years^*a*^

	Total (n = 21)	Improved FIS Points (n = 11)	Did Not Improve FIS Points (n = 10)
Graft type			
Hamstring tendon autograft	16 (76)	8 (73)	8 (80)
Bone–patellar tendon–bone autograft	00 (0)	00 (0)	00 (0)
Quadriceps tendon autograft	1 (5)	00 (0)	1 (10)
Allograft	4 (19)	3 (27)	1 (10)
Multiligamentous injury			
MCL	6 (29)	2 (18)	4 (40)
MCL + posterior cruciate ligament	1 (5)	00 (0)	1 (10)
MCL + patellar tendon	1 (5)	1 (9)	00 (0)
Lateral collateral ligament	1 (5)	1 (9)	00 (0)
None	12 (57)	7 (64)	5 (50)
Chondral injury			
Lateral	8 (38)	4 (36)	4 (40)
Medial	2 (10)	1 (9)	1 (10)
Medial + lateral	00 (0)	00 (0)	00 (0)
None	11 (52)	6 (55)	5 (50)
Meniscal injury			
Lateral	7 (33)	5 (45)	2 (20)
Resection	3 (14)	3 (27)	00 (0)
Repair	4 (19)	2 (18)	2 (20)
Medial	2 (10)	1 (9)	1 (10)
Resection	00 (0)	00 (0)	00 (0)
Repair	2 (10)	1 (9)	1 (10)
Medial + lateral	6 (29)	3 (27)	3 (30)
Resection	3 (14)	2 (18)	1 (10)
Repair	3 (14)	1 (9)	2 (20)
None	6 (29)	2 (18)	4 (40)
Additional surgery (within 3 y after first ACLR)			
Ipsilateral ACLR	2 (10)	2 (18)	00 (0)
Contralateral ACLR	2 (10)	00 (0)	2 (20)
Chondral repair	2 (10)	2 (18)	00 (0)
Other	2 (10)	1 (9)	1 (10)
None	13 (62)	6 (55)	7 (70)

a Data are presented as n (%). ACLR, anterior cruciate ligament reconstruction; FIS, International Ski and Snowboard Federation; MCL, medial collateral ligament.

## Discussion

This study evaluated individual performance outcomes of 30 high-level alpine ski racers after an ACL injury. We analyzed 4 different on-snow performance statistics obtained at 1 year before ACLR and at 3 years after ACLR alongside athlete characteristics and concurrent injuries. Our main finding was that most ski racers returned to the same competition level but that approximately half failed to restore their performance to preinjury values by 3 years after surgery. We found that using performance statistics beyond FIS points gave a more complete picture of performance recovery in World Cup ski racers. However, no association was found between surgical or athlete-specific factors and performance outcomes.

Importantly, 90% of ski racers returned to competition within 3 years after ACLR, confirming previous reports of high rates of return to sport among elite ski racers.^6,12,13^ A retrospective analysis of French national team skiers who suffered ACL ruptures between 1980 and 2003 reported that every skier (n = 148) returned to competition.^13^ In that study, Haida et al^13^ compared performance metrics before and after ACLR at the group level including FIS points, FIS ranking, and medals at World Cups, World Ski Championships, and the Olympics. Better outcomes in these performance metrics were achieved after ACLR. Similarly, Csapo et al^6^ noted high return to competition rates among international ski racers and found significantly better (lower) FIS points at 1 year after the injury, on average, compared with immediately before the injury. Conflicting with these findings, in the present study, 14 ski racers who competed at World Cups showed no differences before and after ACLR in FIS points (7.0 ± 3.9 vs 5.6 ± 3.3, respectively) or FIS ranking (27.4 ± 18.2 vs 21.8 ± 15.7, respectively). In total, there were 9 World Cup medal performances before the ACL injury and 36 World Cup medal performances after the ACL injury, with a single athlete achieving 21 of these medal performances. Skiers competed in more total races after ACLR than before ACLR (243 vs 749, respectively), likely owing to the young age at which most ACL injuries occurred.

On an individual level, among the 30 ski racers evaluated, 16 (53%) were able to achieve lower FIS points in at least one of the speed or technical disciplines by 3 years after ACLR. Similarly, 13 of 30 (43%) ski racers achieved lower FIS ranking in 1 of the 2 disciplines in this time frame. In comparison, in the matched control group of ski racers, 18 of 30 (60%) achieved lower FIS points, and 19 of 30 (63%) attained lower FIS ranking in 1 of the 2 disciplines. After an ACL injury, 3 of the 30 ski racers retired from alpine ski racing, all of whom were aged>25 years at the time of the ACL rupture. Interestingly, while most of the ski racers competed in both disciplines in the year before the injury, 5 athletes competed in exclusively technical events after ACLR, and 6 athletes competed in exclusively speed events after ACLR.

We calculated and examined 2 additional performance statistics—average placing and average percentage behind the winning time—to provide a more comprehensive picture of performance. Importantly, the discipline and competition category must be accounted for when comparing these 2 metrics. Among ski racers competing in the World Cup speed discipline before an ACL rupture, 6 of 12 (50%) had better average placing, and 5 of 12 (42%) had a better average percentage behind the winning time in the third year after surgery. In the technical discipline, 2 of the 6 (33%) athletes competing in World Cup events exhibited better performance in both average placing and average percentage behind the winning time. Notably, 3 of the ski racers in each of the comparisons above, who were classified as not showing an improvement in performance, retired after surgery. These additional performance statistics may provide alternative or complementary insights on FIS points and FIS ranking, particularly when these outcomes are contrasting (as can be seen for the speed discipline in Table 2).

### Influence of Age on Return to Performance

The mean age at the time of the first ACL rupture was 21.6 ± 3.3 years, aligning closely with the results of Haida et al,^13^ who reported a mean age of 21.1 ± 4.0 years for initial ACL tears among skiers representing the French national team. Haida et al^13^ found that female athletes suffered ACL tears at a younger age than male athletes (19.9 ± 3.5 vs 22.6 ± 4.1 years, respectively; *P* < .0001). In our study, women suffered ACL tears at a mean 20.5 ± 3.6 years of age, while men experienced ACL tears at a mean 23.2 ± 3.0 years of age (*P* = .06).

Age at the time of injury appears to be predictive of return to sport, with younger athletes (<25 years) having higher rates of return.^9,29^ Less has been documented regarding the effect of age at the time of ACL rupture on performance after ACLR. We found that the mean age of ski racers at the time of ACL rupture who achieved lower FIS points after surgery was similar to that of those who did not (21.3 ± 3.5 vs 22.6 ± 3.6 years, respectively; *P* = .33), suggesting that age at the time of ACL rupture did not influence a ski racer’s likelihood of improving performance within 3 years of surgery. These results are in contrast to those reported by Haida et al^13^ in which French national team ski racers who improved FIS points after ACLR were significantly younger than their counterparts who did not. Perhaps our observations would be similar to those of Haida et al^13^ if we were to extend the duration of our postoperative observation period.

Typically, the age at peak performance for international ski racers is reported to be between 25 and 28 years.^13,27^ Indeed, in our analysis of all injured and uninjured ski racers who competed at World Cup events between 2005 and 2023, peak performance (as determined by FIS points) was achieved at the mean age of 25.5 ± 2.7 years. Interestingly, in our group of ski racers with ACL injuries, 8 of 30 were aged >25 years at the time of ACL rupture, and only 2 (25%) of these ski racers improved FIS points by 3 years.

### Concurrent Injuries and Return to Performance

In the current study, nearly half (43%) of the ski racers sustained multiligamentous injuries, with all but one of these injuries involving the ipsilateral medial collateral ligament (MCL). This pattern of concomitant ACL and MCL injuries in skiers is common. MCL injuries occurring alongside ACL ruptures have previously been reported in 11% of competitive ski racers^10^ and in 31% to 50% of recreational skiers.^8,22,23^

We also observed that a high proportion (71%) of ACL injuries occurred with concomitant meniscal tears and that the lateral meniscus was the most often affected. Only 13% of all meniscal tears were isolated to the medial meniscus. Skiers commonly exhibit a higher proportion of lateral than medial meniscal tears, as reported in several studies,^6,8,17,22^ although not all.^23^ Similarly, most chondral lesions (80%) in the present study occurred in the lateral compartment.

The relationship between concurrent injuries and performance outcomes in ski racers after ACLR has not been previously reported. In our cohort, 4 of the 9 ski racers (44%) who sustained multiligamentous injuries improved FIS points by 3 years after surgery. Similarly, 9 of 15 (60%) ski racers who sustained meniscal tears and 5 of 10 (50%) ski racers who sustained chondral lesions improved FIS points by 3 years after surgery. This suggests that approximately half of ski racers with concomitant injuries can experience improved performance within 3 years.

Among the 3 athletes who retired from racing after ACLR, 1 suffered a multiligamentous injury (ACL + MCL), accompanied by grade 3 chondral surface damage and a complex lateral meniscal tear. Another athlete suffered a multiligamentous injury (ACL + MCL + posterior cruciate ligament) along with grade 1 lateral condyle chondral surface damage. The third athlete, while retiring from alpine ski racing, transitioned to competing in ski cross after suffering an isolated ACL tear with grade 1 chondral surface damage.

Graft type may also influence performance outcomes, as different reconstruction techniques result in distinct comorbidities and neuromuscular deficits to the knee extensors and knee flexors. In our study, 24 of 30 (80%) athletes received hamstring tendon autografts, which is typical for ski racers.^7,10,12,16^ Ski racing involves sustained highly forceful eccentric/quasi-isometric knee extensor muscle actions,^4^ and the use of hamstring tendon autografts is likely a consideration to reduce the incidence of anterior knee pain and the persistent knee extensor deficits common with bone–patellar tendon–bone and quadriceps tendon autografts.^11,25^

### Limitations

A challenge inherent to assessing elite sporting populations such as alpine ski racers is dealing with small sample sizes. Nevertheless, given the high risk of traumatic ACL injuries/reinjuries in alpine ski racing, the information gleaned from these types of investigations is important for practitioners and sport organizations for managing the return to performance process. We conducted a descriptive analysis of preinjury and postinjury performance without considering the athletes’ preinjury performance trajectory, and therefore, no causation can be inferred. Skiers may have also suffered time-loss injuries other than ACL ruptures during the period of observation that are not accounted for. Further, we did not report information on the career length of skiers after ACLR.

### Perspective

Understanding sport-specific performance outcomes and their timelines after an ACL injury is important for planning the rehabilitation process and aligning stakeholder expectations. We showed that most ski racers returned to their preinjury level of competition, but the athletes demonstrated substantial individual variability in the restoration of their on-snow performance. We also highlight the importance of incorporating several sport-specific performance statistics in an analysis of outcomes to provide a broader characterization of performance. Based on our results, it can be expected that only half of competitive ski racers who sustain an ACL injury will improve their performance by 3 years after surgery.

## Conclusion

Approximately half of competitive ski racers achieved better performance by 3 years after ACLR compared with before the ACL injury. Concomitant injuries to the lateral meniscus and lateral chondral surface were common in ski racers with ACL injuries but did not seem to be associated with the likelihood of achieving better postinjury performance.

## References

[1] Ardern CL Taylor NF Feller JA Webster KE. Fifty-five percent return to competitive sport following anterior cruciate ligament reconstruction surgery: an updated systematic review and meta-analysis including aspects of physical functioning and contextual factors. Br J Sports Med. 2014;48(21):1-11. 25157180 10.1136/bjsports-2013-093398

[2] Barth KA Lawton CD Touhey DC , et al. The negative impact of anterior cruciate ligament reconstruction in professional male footballers. Knee. 2019;26(1):142-148. 30449615 10.1016/j.knee.2018.10.004

[3] Bere T Flørenes TW Nordsletten L Bahr R. Sex differences in the risk of injury in World Cup alpine skiers: a 6-year cohort study. Br J Sports Med. 2013;48(1):36-40. 23673520 10.1136/bjsports-2013-092206

[4] Berg HE Eiken O Tesch PA. Involvement of eccentric muscle actions in giant slalom racing. Med Sci Sports Exerc. 1995;27(12):1666-1670. 8614323

[5] Chen JW Maldonado DR Kowalski BL , et al. Best practice guidelines for propensity score methods in medical research: consideration on theory, implementation, and reporting. A review. Arthroscopy. 2022;38(2):632-642. 34547404 10.1016/j.arthro.2021.06.037

[6] Csapo R Hoser C Gföller P Raschner C Fink C. Fitness, knee function and competition performance in professional alpine skiers after ACL injury. J Sci Med Sport. 2019;22:S39-S43. 10.1016/j.jsams.2018.06.014 29980379

[7] Csapo R Runer A Hoser C Fink C. Contralateral ACL tears strongly contribute to high rates of secondary ACL injuries in professional ski racers. Knee Surg Sports Traumatol Arthrosc. 2021;29(6):1805-1812. 32804249 10.1007/s00167-020-06234-8

[8] Duncan JB Hunter R Purnell M Freeman J. Meniscal injuries associated with acute anterior cruciate ligament tears in alpine skiers. Am J Sports Med. 1995;23(2):170-172. 7778701 10.1177/036354659502300208

[9] Edwards PK Ebert JR Joss B , et al. Patient characteristics and predictors of return to sport at 12 months after anterior cruciate ligament reconstruction: the importance of patient age and postoperative rehabilitation. Orthop J Sports Med. 2018;6(9):2325967118797575. 10.1177/2325967118797575 PMC6149022 30263898

[10] Farinelli L Csapo R Meena A Abermann E Hoser C Fink C. Concomitant injuries associated with ACL rupture in elite professional alpine ski racers and soccer players: a comparative study with propensity score matching analysis. Orthop J Sports Med. 2023;11(8):23259671231192127. 10.1177/23259671231192127 PMC10467387 37655251

[11] Feller JA Webster KE. A randomized comparison of patellar tendon and hamstring tendon anterior cruciate ligament reconstruction. Am J Sports Med. 2003;31(4):564-573. 12860546 10.1177/03635465030310041501

[12] Guy S Fayard JM Saithna A , et al. Risk of graft rupture after adding a lateral extra-articular procedure at the time of ACL reconstruction: a retrospective comparative study of elite alpine skiers from the French national team. Am J Sports Med. 2022;50(6):1609-1617. 35416071 10.1177/03635465221085027

[13] Haida A Coulmy N Dor F , et al. Return to sport among French alpine skiers after an anterior cruciate ligament rupture: results from 1980 to 2013. Am J Sports Med. 2016;44(2):324-330. 26598331 10.1177/0363546515612764

[14] Harris JD Erickson BJ Bach BR , et al. Return-to-sport and performance after anterior cruciate ligament reconstruction in National Basketball Association players. Sports Health. 2013;5(6):562-568. 24427434 10.1177/1941738113495788 PMC3806178

[15] Jordan MJ Aagaard P Herzog W. Anterior cruciate ligament injury/reinjury in alpine ski racing: a narrative review. Open Access J Sport Med. 2017;8(2):71-83. 10.2147/OAJSM.S106699 PMC5386612 28435336

[16] Jordan MJ Aagaard P Herzog W. Rapid hamstrings/quadriceps strength in ACL-reconstructed elite alpine ski racers. Med Sci Sports Exerc. 2015;47(1):109-119. 24824771 10.1249/MSS.0000000000000375

[17] Jordan MJ Doyle-Baker P Heard M Aagaard P Herzog W. A retrospective analysis of concurrent pathology in ACL-reconstructed knees of elite alpine ski racers. Orthop J Sports Med. 2017;5(7):2325967117714756. 10.1177/2325967117714756 PMC5528939 28812037

[18] Jordan MJ Morris N Lane M , et al. Monitoring the return to sport transition after ACL injury: an alpine ski racing case study. Front Sports Act Living. 2020;2:12. 33345007 10.3389/fspor.2020.00012 PMC7739580

[19] Mai HT Chun DS Schneider AD , et al. Performance-based outcomes after anterior cruciate ligament reconstruction in professional athletes differ between sports. Am J Sports Med. 2017;45(10):2226-2232. 28510477 10.1177/0363546517704834

[20] Mohtadi NG Chan DS. Return to sport-specific performance after primary anterior cruciate ligament reconstruction: a systematic review. Am J Sports Med. 2018;46(13):3307-3316. 29028445 10.1177/0363546517732541

[21] Niederer D Engeroff T Wilke J Vogt L Banzer W. Return to play, performance, and career duration after anterior cruciate ligament rupture: a case-control study in the five biggest football nations in Europe. Scand J Med Sci Sport. 2018;28(10):2226-2233. 10.1111/sms.13245 29927499

[22] Paletta GA Levine DS O’Brien SJ Wickiewicz TL Warren RF Johnson R. Patterns of meniscal injury associated with acute anterior cruciate ligament injury in skiers. Am J Sports Med. 1992;20(5):542-547. 1443322 10.1177/036354659202000510

[23] Posch M Schranz A Lener M Tecklenburg K Burtscher M Ruedl G. In recreational alpine skiing, the ACL is predominantly injured in all knee injuries needing hospitalisation. Knee Surg Sports Traumatol Arthrosc. 2021;29(6):1790-1796. 32803275 10.1007/s00167-020-06221-z PMC8126542

[24] Rosenbaum PR Rubin DB. The central role of the propensity score in observational studies for causal effects. Biometrika. 1983;70(1):41-55.

[25] Shaieb MD Kan DM Chang SK Marumoto JM Richardson AB. A prospective randomized comparison of patellar tendon versus semitendinosus and gracilis tendon autografts for anterior cruciate ligament reconstruction. Am J Sports Med. 2002;30(2):214-220. 11912091 10.1177/03635465020300021201

[26] Shrier I Stokes T Wang C Trejovargas J Impellizzeri FM Steele RJ. Investigating the effect of return-to-play timing after injury on performance: does the analysis answer the research objective? Sports Med. 2023;53(5):949-958. 36378413 10.1007/s40279-022-01792-y

[27] Steidl-Müller L Hildebrandt C Raschner C Müller E. Challenges of talent development in alpine ski racing: a narrative review. J Sports Sci. 2019;37(6):601-612. 30676888 10.1080/02640414.2018.1513355

[28] Tramer JS Khalil LS Ziedas A Mehran N Okoroha KR. Return to play and performance in the Women’s National Basketball Association after anterior cruciate ligament reconstruction. Orthop J Sports Med. 2020;8(9):2325967120947078. 10.1177/2325967120947078 PMC7498979 32984422

[29] Webster KE Feller JA. Return to level I sports after anterior cruciate ligament reconstruction: evaluation of age, sex, and readiness to return criteria. Orthop J Sports Med. 2018;6(8):2325967118788045. 10.1177/2325967118788045 PMC6088492 30116761

[30] Yung KK Ardern CL Serpiello FR Robertson S. A framework for clinicians to improve the decision-making process in return to sport. Sports Med Open. 2022;8(1):52. 35416633 10.1186/s40798-022-00440-z PMC9008084

